# Overexpression of ANXA1 in Penile Carcinomas Positive for High-Risk HPVs

**DOI:** 10.1371/journal.pone.0053260

**Published:** 2013-01-14

**Authors:** Marilia Freitas Calmon, Mânlio Tasso de Oliveira Mota, Érica Babeto, Natália Maria Candido, Ana Paula Girol, Carlos Fabian Mendiburu, Jane Lopes Bonilha, Rodrigo Vellasco Duarte Silvestre, Bruno Miziara Rosa, Jorge Alberto Thomé, Gustavo Hernandez Américo Medeiros, Fernando Augusto Soares, Gustavo Cardoso Guimarães, José Germano Ferraz de Arruda, Sonia Maria Oliani, Luisa Lina Villa, José Vassallo, Paula Rahal

**Affiliations:** 1 São Paulo State University, São José do Rio Preto, São Paulo, Brazil; 2 Institute of Anatomical Pathology and Cytopathology, São José do Rio Preto, São Paulo, Brazil; 3 College of Medicine of Rio Preto,São José do Rio Preto, São Paulo, Brazil; 4 University Hospital João de Berros Barreto, Belem, Pará, Brazil; 5 Hospital A. C. Camargo,São Paulo, São Paulo, São Paulo, Brazil; 6 Department of Radiology and Basic Oncology, School of Medicine, University of São Paulo, and College of Medical Sciences of Santa Casa de São Paulo, São Paulo,São Paulo, Brazil; Ohio State University Medical Center, United States of America

## Abstract

The incidence of penile cancer varies between populations but is rare in developed nations. Penile cancer is associated with a number of established risk factors and associated diseases including phimosis with chronic inflammation, human papillomavirus (HPV) infection, poor hygiene and smoking. The objective of this study was to identify genes related to this type of cancer. The detection of HPV was analyzed in 47 penile squamous cell carcinoma samples. HPV DNA was detected in 48.9% of penile squamous cell carcinoma cases. High-risk HPV were present in 42.5% of cases and low-risk HPV were detected in 10.6% of penile squamous cell carcinomas. The *RaSH* approach identified differential expression of *Annexin A1* (*ANXA1), p16, RPL6, PBEF1* and *KIAA1033* in high-risk HPV positive penile carcinoma; *ANXA1* and *p16* were overexpressed in penile squamous cells positive for high-risk HPVs compared to normal penile samples by qPCR. ANXA1 and p16 proteins were significantly more expressed in the cells from high-risk HPV-positive penile carcinoma as compared to HPV-negative tumors (*p*<0.0001) independently of the subtype of the carcinoma. Overexpression of *ANXA1* might be mediated by HPV E6 in penile squamous cell carcinoma of patients with high-risk HPVs, suggesting that this gene plays an important role in penile cancer.

## Introduction

Penile cancer affects predominantly men aged between 50 and 70 years [1]–[3]. Penile cancer is associated with several established risk factors and associated diseases including phimosis with chronic inflammation, human papillomavirus (HPV) infection, poor hygiene and smoking [4]. Studies reported an overall HPV prevalence of, approximately, 48% in penile cancer worldwide [5], [6]. In penile carcinomas the most common HPV types are HPV 16 and HPV 18. HPV 16 is most prevalent in North America, Europe, South America and India [5], [7].

HPV contributes to tumorigenesis predominantly through the action of viral oncoproteins (E6 and E7) [8]. E6 inhibits apoptotic signaling in response to growth-suppressive cytokines by interacting with tumor necrosis factor (TNF)-α receptor TNFR1, FAS-associated protein with death domain (FADD) and caspase 8, and via degradation of pro-apoptotic BAX and BAK. E6-mediated degradation of PDZ proteins leads to a loss of cell polarity and induces hyperplasia [9], [10]. Therefore, E6 can interfere with the regulation of expression of genes by interacting with and binding to the proteins mentioned above, prompting the interest of some researchers in identifying new genes whose expression can be disturbed by E6 protein.

One gene that could be subject to regulation by the oncoprotein E6 is ANXA1 as previously suggested by Shimoji et al., 2009 [11]. The annexin superfamily proteins have been implicated in several cellular processes including differentiation, apoptosis, proliferation and inflammation. The expression of *ANXA1* has been studied in various types of cancer, but there is no consensus concerning the role this protein plays during tumor initiation and/or progression. Some studies observed a correlation between the decrease of *ANXA1* mRNA and protein with esophageal, prostate and breast cancers [12]–[15]. On the contrary, others studies showed the overexpression of *ANXA1* in head and neck and pancreatic cancers [16], [17].

Here, we aimed to identify novel genes differentially expressed in penile squamous cell carcinoma positive for high-risk HPVs and evaluate a possible correlation between HPV positivity, the expression of the genes and the subtypes of penile squamous cell carcinoma.

## Materials and Methods

### Patients

Archival paraffin wax-embedded tissue sections from 47 penile squamous cell carcinoma were obtained and reviewed by a pathologist, with approval from the Research Ethics Committee of the College of Medicine of São José do Rio Preto, Research Ethics Committee of Hospital A. C. Camargo, São Paulo, and Research Ethics Committee of University Hospital João de Barros, Belem, all located in Brazil. Twelve penile squamous cell carcinoma samples and seven normal penile fresh-frozen tissue samples were obtained from the College of Medicine of São José do Rio Preto, and Hospital A. C. Camargo. The use of patient-derived material was approved by the institution's Committee Research Ethics Board and written consent was obtained from all patients. Tissues were obtained at surgery from patients undergoing tumor resection, and the diagnosis of penile squamous cell carcinoma was verified post-operatively using histopathology. All slides were histologically examined accordingly to the TNM classification system (American Joint Comittee on Cancer) [18]. The slides were also classified according to morphologic criteria outlined in the Atlas of Tumor Pathology [19]. The following variants were considered: usual, basaloid, warty, papillary, verrucous, sarcomatoid and mixed squamous cell carcinoma.

### DNA Extraction

DNA was extracted from 6 slices of 10 micra of paraffin wax-embedded sections using the QIAamp DNA FFPE Tissue kit (Cat. No. 56404; Qiagen, Crawley, U.K.). The polymerase chain reaction (PCR) was performed on DNA extracted from penile squamous cell carcinoma samples. Purified DNA (1–10%) was subjected to PCR. The amplification of a fragment of the *β-globin* gene served as an internal control to assess the sufficiency of DNA in each specimen.

### HPV DNA Detection

*Globin* positive specimens were analyzed by PCR for the presence of HPV DNA using the consensus primers GP5+/GP6+, which flank a fragment of approximately 140 bp of the *L1 gene*, a highly conserved sequence in HPV genomes, allowing several genital HPV types to be detected [20] The reaction components in a final volume of 50 ul were: 1.0 mM GP5+/GP6+; 2.0U Taq DNA polymerase (Fermentas, California, USA); 20 mM Tris–HCl, pH 8.4; 50 mM KCl; 3.0 mM MgCl_2_; 200 mM of each deoxyribonucleotide (Amersham Pharmacia Biotech, New Jersey, USA) and between 3.0 and 7.0 µl of DNA from the samples. The PCR conditions were an initial step of five min at 94°C, 40 cycles of one min at 94°C, one min at 45°C, and 90 s at 72°C; the last cycle was five min at 72°C. For each reaction, DNA from HeLa cells, a HPV-18 positive cervical cancer derived cell line, was used as a positive control and water and DNA from C33 cells were used as negative controls. The C33 and HeLa cell lines were a generous gift from Dr. Luisa Lina Villa from University of São Paulo [21], [22].

### HPV Genotyping by INNO-LiPA

Genotyping was performed with the INNO-LiPA HPV Genotyping Extra test (Innogenetics, Gent, Belgium) allowing the identification of 28 different HPV genotypes as well as the HLA-DPB1 gene as internal control for DNA quality. As recommended by the manufacturer, only samples positive for any HPV and/or for the HLA-DPB1 gene were included in the analysis.

### RNA Extraction and RT-PCR

Total RNA was isolated from penile squamous cell carcinoma tissue and normal tissue using TRIzol reagent (solution for extraction of RNA, Life Technologies, Grand Island, USA) according to the manufacturer’s instructions. RNA integrity post-purification was ensured using the Agilent 2100-Bioanalyser, giving a minimal RIN value of 5.5.

### Rapid Subtractive Hybridization (*RaSH*)

Four fresh-frozen samples of penile squamous cell carcinoma were used to perform RaSH methodology. Tissues adjacent to tumor and tumor tissues from the same patient were reviewed by two pathologists and microdissected aiming to obtain most representative tumoral and morphologically normal tissues. HPV 16 was detected in tumoral cells while normal samples were HPV DNA negative. *RaSH* cDNA libraries were performed as described previously [23], with modifications. From the 25 µg total RNA pool, cDNAs were synthesized and digested with MboI (Invitrogen Life Technologies, California, USA) at 37°C for one hour and extracted with phenol-chloroform followed by ethanol precipitation. The digested cDNAs were mixed with 20 mmol/L of the primers XDPN-14 (5′CTGATCACTCGAGA3’) and XDPN-12 (5′GATCTCTCGAGT3’) in 30 µL of 1X T4 DNA Ligase Buffer (Invitrogen Life Technologies, California, USA), heated at 55°C for one min, and cooled to 14°C within one hour. Ligation was carried out overnight at 14°C after adding nine units of T4 DNA ligase to each sample.

The samples were diluted to 100 µl and 40 ul of the mixture was used for PCR amplification with the primer XDPN-18 (5′CTGATCACTCGAGAGATC 3′). Aliquots (10 µg) of the tester PCR products (penile carcinoma or normal tissue) were digested with 20 units of XhoI (Invitrogen Life Technologies, California, USA) and purified with phenol-chloroform extraction and ethanol precipitation. The fragments were inserted into XhoI-digested pZERO plasmid (1 µg/µl) at 16°C for three hours. The constructs were introduced into TOP10 competent cells. Two *RaSH* cDNA libraries were prepared, one using cDNA from the penile squamous cell carcinoma as a tester and normal tissue of penis as a driver, and the other using cDNA from normal tissue of penis as a tester with cDNA from the penile squamous cell carcinoma as a driver.

Bacterial colonies were analyzed using PCR and the M13 forward and M13 reverse primers to identify those with an insert. The sequences of these clones were determined using a DNA sequencer (ABI PRISM 377, Applied Biosystems, California, USA) and DYEnamic ET Dye Terminator Sequencing Kit (Amersham Biosciences, New Jersey, USA). A total of 230 cDNA clones were sequenced, 27 clones obtained from the reverse library (downregulated genes) and 30 clones obtained from the upregulated genes library. The sequences were analyzed using an annotation pipeline with four steps: (1) quality checking, phred base-calling, cutoff 0.09, minmatch 10 and minscore 20; (2) vector trimming and removal of undesirable sequences such as bacterial, mitochondrial and rRNA sequences; (3) masking of repetitive elements and screening of low-complexity regions by Repeat Masker, using the default settings [24]; (4) annotation against existing databases, using BLASTN with default parameters. Significant hits were determined using an E-value threshold of 10−15 for searches against nucleotide sequence databases [25].

### qPCR

qPCR was used to assess the expression of genes identified by rapid subtraction hybridization (*RaSH*) in fresh samples of penile squamous cell carcinoma. For qPCR, 12 fresh samples of penile squamous cell carcinoma positive for high-risk HPVs and a pool of 7 fresh normal penile tissue samples were used; the normal tissues were defined as the normal reference. Gene-specific primers for qPCR were designed for optimal hybridization kinetics using the Primer 3.0 program (provided by the Whitehead/MIT Center for Genome Research, Cambridge, MA).

Quantitative Real-time PCR was performed using an ABI prism 7300 sequencer detector system and SybrGreen PCR Core Reagent (Applied Biosystems, California, USA), following the manufacturer’s protocol. In brief, the reaction mixture (20 µl total volume) contained 25 ng cDNA, gene-specific forward and reverse primers for each gene, and 10 µL of 2x Quantitative Sybr Green PCR Master Mix (Applied Biosystems, California, USA). Relative quantification was given by the CT values, determined for triplicate reactions of penile tumor samples and reference samples for each gene and tubulin (*TUBA1A*) for the endogenous control. The primer sequences are available on request.

Therefore, the relative expression of each specific gene was calculated by using the formula: R = (E target)^ΔCt target (control - sample)^/(E endogenous)^ΔCt endogenous (control - sample)^, as previously described [26]. The cut-off for analysis of gene expression was ≥4 for increases and decreases in expression. A value below this cut-off was considered to indicate that the increase/decrease in expression was not significant.

### Immunohistochemistry

For histopathological evaluation, two observers that were unaware of the clinical data, reviewed independently the slides, and discrepancies were resolved by joint review of the slides in question. The primary lesion was staged according to the TNM classification system (Americam Joint Committee on Cancer) [18].

Immunohistochemistry was used to evaluate *ANXA1* and *p16* protein expressions in 20 histologically normal tumor margins (10 margins from squamous cell carcinoma of penis high-risk HPV positive samples and 10 margins from squamous cell carcinoma of penis HPV negative samples - control group), 24 squamous cell carcinoma of penis samples without HPV (HPV-negative group), 3 samples of squamous cell carcinoma of penis samples with low-risk HPVs (HPV-low risk group) and 20 squamous cell carcinoma of penis samples positive for high-risk HPVs (HPV-high risk group) (Table 1).

**Table 1:** Description of penile squamous cell carcinoma patients with clinical parameters and HPV types.

Variable	Number of patients
**Age years (median 67)**	
≤67	24
>67	23
**T stage**	
T_1a,1b, 2_	42
T_3,4_	5
**N stage**	
N_0,1_	45
N_2,3_	2
**M stage**	
M_0_	47
M_1_	0
**HPV Types**	
None	24
11	3
16	18
16,11	1
35,11	1

The detection of ANXA1 and p16 were conducted in 4 µm sections of each designated formalin-fixed, paraffin-embedded tissue blocks. After an antigen retrieval step using citrate buffer pH 6.0, the endogenous peroxide activity was blocked and the sections were incubated overnight at 4°C with the primary antibodies: monoclonal anti-p16 (1∶1000) (Abcam, Cambridge, UK) or rabbit polyclonal anti-ANXA1 (1∶2000) (Zymed Laboratories, Cambridge, UK) diluted in 1% BSA. After washing, sections were incubated with a secondary biotinylated antibody (Dako, Cambridge, UK). Positive staining was detected using a peroxidase conjugated streptavidin complex and colour developed using DAB substrate (Dako, Cambridge, UK). The sections were counterstained with hematoxylin.

The ANXA1 and p16 densitometric analyses were conducted with an Axioskop II microscope (Zeiss, Germany) using the Software Axiovision™ (Zeiss). For these analyses five different fields from each tumor fragments were used and 20 different points were analyzed for an average related to the intensity of immunoreactivity. The values were obtained as arbitrary units (a.u.).

### Statistical Analysis

Statistical analysis was performed using GraphPad Prism 6 software (GraphPad, California, USA) and data were expressed as means ± SEM. The Mann-Whitney U test was used to assess differences in age. The Wilcoxon Signed Ranks Test was applied to compare the gene expression levels in tumor tissue and normal penile tissue. Data from protein expression detected by immunohistochemistry were statistically examined by Kruskal-Wallis with Tukey’s *post hoc* tests for multiple comparisons. The significance level was set at *P*<0.05 for all analyses.

## Results

### Pathological Findings and HPV Detection

The presence of penile squamous cell carcinoma was confirmed in all samples analyzed using a histopathological revision examination; these samples were subjected to DNA extraction for molecular analysis. All fresh samples were positive for the amplification of a human β-globin gene.

The patient age range was 31 to 95 years (mean 63 years), with no differences between patients with penile squamous cell carcinoma HPV positive and HPV negative (*p* = 0.70). HPV DNA was present in 23 of 47 (48.9%) penile squamous cell carcinoma cases studied. Most commonly only 1 genotype was identified [21 of 23 (91.3%)]. High-risk HPVs were present in 42.5% (20/47) of the cases and low-risk HPVs were identified in 10.6% (5/47) of penile squamous cell carcinoma samples. High-risk type 16 was the most prevalent type, present in 19 of 23 (82.6%) of HPV positive tumours. HPV18 was not detected. In the majority of HPV-positive tumours [18 of 23 (78.2%)] HPV16 was the only HPV type detected. In tumors with multiple viral infections there was a simultaneous presence of low-risk and high-risk HPV (Table 1).

The usual subtype of penile squamous cell carcinoma was the most common subtype present in 83% of the cases, followed by verrucous (8.5%), warty (4.2%), papillary (2.1%) and sarcomatoid (2.1%). For the usual type, HPV DNA was detected in 19 of 39 (48.7%) tumours, with high-risk HPV16 present in 15 of 39 (38.5%) samples. Verrucous and warty subtypes were positive for HPV DNA in 50% of the analyzed samples, with HPV16 present in the HPV positive samples. HPV type 16 was detected in 100% of the papillary tumours. In contrast, HPV was not detected in sarcomatoid tumours. No association of any of the HPV genotypes with subtypes of penile squamous cell carcinoma was found (Table 2).

**Table 2:** Histological Subtypes of penile squamous cell carcinoma and HPV Genotypes.

Subtype	11	16	35 and 11	16 and 11	Negative	Total
**Usual**	3	14	1	1	20	39
**Verrucous**		2			2	4
**Warty**		1			1	2
**Sarcomatoid**					1	1
**Papillary**		1				1
**Total**	3	18	1	1	24	47

### Identification of Genes Differentially Expressed in Penile Squamous Cell Carcinoma by RaSH

The *RaSH* approach was adopted to identify genes expressed differentially in penile with high-risk HPVs. After alignment with the RefSeq database, sequences that presented >90% of the target sequence length at alignment were selected. These included *ANXA, p16, RPL6, PBEF1* and *KIAA1033.*

### Validation of Identified Genes by qPCR

For the detection of genes expressed differentially in penile tumors, a gene expression profile was performed using 12 fresh samples of primary penile squamous cell carcinoma positive for high-risk HPVs. The relative expression levels of five genes were compared using qPCR, using triple determination and normalization based on the *tubulin* level. In the evaluation of the target genes, penile squamous cell tumor samples were used, and a pool of normal penile tissues was used as a reference (control group).

**Figure 1:**
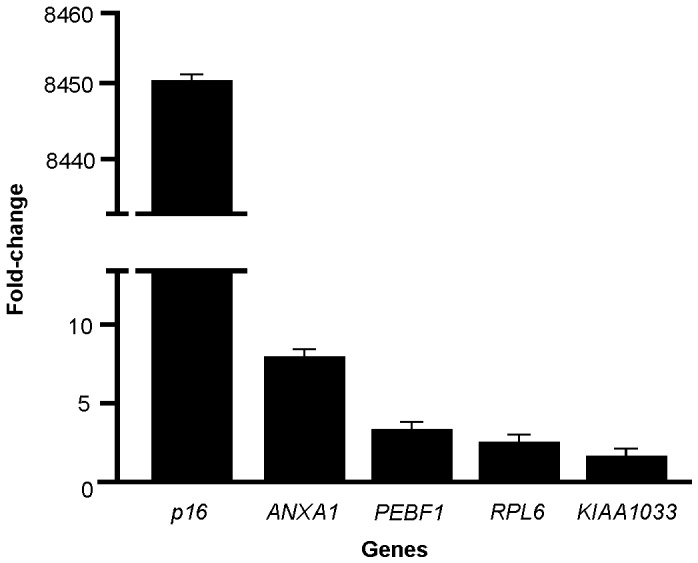
Relative expression media of the selected genes for validation using qPCR.

The expression of the genes *PBEF1, KIAA1033* and *RPL6* did not differ between penile squamous cell carcinoma and normal penile tissue, with fold-change values for gene expression raging from 1.6 to 3.3. *ANXA1* and *p16* were overexpressed in penile squamous cell carcinoma samples compared with the sample reference (*P = *0.002 and 0.0001 respectively) and the fold-change values for gene expression were 7.9 and 8450, respectively (Figure 1). The results obtained for *ANXA1* and *p16* using qPCR were in agreement with the *RaSH* method, providing further evidence that these genes are cancer-related.

### Immunohistochemistry

**Figure 2:**
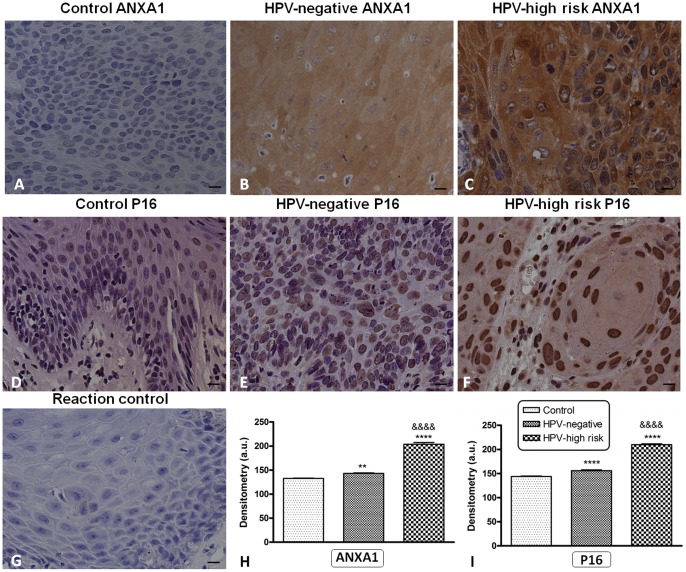
Immunolocalization of annexin A1 (ANXA1) and p16 in human primary penile squamous cell carcinoma and histologically normal tumor margins. ANXA1 immunostaining in A) Histologically normal tumor margins; B) Human primary penile squamous cell carcinoma HPV-negative; C) Human primary penile squamous cell carcinoma positive for high-risk HPV. p16 immunostaining in D) Histologically normal tumor margins; E) Human primary penile squamous cell carcinoma HPV-negative; F) Human primary penile squamous cell carcinoma positive for high-risk HPV. G) Reaction control for ANXA1. H) Graphic of densitometry of the immunostaining of ANXA1 in the samples analyzed. I) Graphic of densitometry of the immunoistaining of p16 in the samples analyzed. Bars = 50 µm. (** = *p*<0.01; **** = *p*<0.0001; &&&& = *p*<0.0001, Tukey’s *post hoc* test).

Immunostaining of ANXA1 was mostly weak or negative in the cytoplasm of cells from tumor margins (control group) (Figure 2A) compared to the samples from the HPV-negative (*p*<0.01, Tukey’s *post hoc* test) (Figure 2B and H) and HPV-high-risk (*p*<0.0001, Tukey’s *post hoc* test) (Figure 2C and H) groups. Low-risk HPV positive squamous cell carcinoma of penis showed decreased expression of ANXA1 compared to the high-risk HPV tumours (data not shown for low-risk HPV positive samples and they were not included in the statistical analysis due to the small number). ANXA1 immunostaining was significantly increased in the cytoplasm of cells from penile squamous cell carcinoma with high-risk HPVs independently of the subtype compared to HPV-negative penile squamous cell carcinoma (*p*<0.0001, Tukey’s *post hoc* test) (Figure 2B, C and H). Immunoreactivity for p16 was not detected or presented a weak expression in the nuclei of the non-neoplastic epithelia (control group) (Figure 2D) and increased immunoreactivity was observed in the nuclei of penile squamous cell carcinoma samples negative for HPV (*p*<0.0001, Tukey’s *post hoc* test) (Figure 2E and I) compared to non-neoplastic epithelia. Low-risk HPV positive penile squamous cell carcinoma samples showed decreased expression of p16 compared to the high-risk HPV penile squamous cell carcinoma samples (data not shown for low-risk HPV positive samples and they were not included in the statistical analysis due to the small number). The penile squamous cell carcinoma samples with high-risk HPVs showed increased p16 expression observed both in the nuclei and in the cytoplasm indenpendently of the subtype (*p*<0.0001, Tukey’s *post hoc* test) (Figure 2F and I) relative to penile squamous cell carcinoma without HPV. Negative control reactions were used for ANXA1 and p16 immunostaining (Figure 2G). ANXA1 and p16 immunodetection showed no significant difference between histological subtypes of penile squamous cell carcinoma since the most prevalent subtype was usual carcinoma (83%).

## Discussion

Overexpression of *ANXA1* mRNA and Annexin-I (ANXA1) protein were detected in squamous cell carcinoma of penis. *ANXA1* was the first member characterized of the annexin superfamily, characterized by the calcium-dependent ability to bind phospholipids. ANXA1 inhibits the activity of cytosolic phospholipase A2 (cPLA2) and cyclooxygenase-2 (COX-2), thus exhibiting anti-inflammatory, anti-pyretic and anti-hyperalgesic activities [27], [28]. In addition, ANXA1 is associated with various physiological processes including cellular differentiation [29], cell proliferation and signal transduction [30], [31]. Furthermore, deregulation of ANXA1 has been correlated with tumor progression in several types of cancer [16], [17], [32]–[39]. One study suggested that ANXA1 appears to be induced in tumor endothelium, and the lack of ANXA1 in ANXA1-KO mice may impair tumor-induced angiogenesis with reduced blood supply explaining retarded tumor growth and metastasis in Lewis Lung carcinoma [40]. Other recent investigation showed that strong cellular and cell surface expression of ANXA1 in tumor cells at the invasion front was significantly associated with the occurrence of metastasis in penile cancer [41]. This finding could be explained by the important role of ANXA1 in regulation of cell invasion and migration. These data corroborate our results that have shown ANXA1 overexpression in all penile squamous cell carcinoma samples analyzed and classified pathologically as stage T3 or T4. Probably, when ANXA1 is expressed, tumors develop more blood vessels and, in consequence, tumors grow faster, suggesting that ANXA1 is a key regulator of pathological angiogenesis and physiological angiogenic balance.

Furthermore, it is the first time in the literature that ANXA1 protein overexpression is associated with HPV related penile cancer. It is known that E6AP binds to ANXA1 *in vivo* and *in vitro* and overexpression of E6AP enhances proteasomal degradation of ANXA1 *in vivo* [11]. Physical and functional association of E6AP with viral proteins, such as HPV16E6 [42] and HCV core protein [43], have also been demonstrated. E6 interaction with E6AP has been reported to be important for skin carcinogenesis in transgenic mouse models [44], [45]. it is possible that the viral proteins such as HPV16E6 redirect E6AP away from ANXA1, which increases increasing the stability of ANXA1, and thereby contributes to viral pathogenesis [11]. Our work also corroborated with this hypothesis since ANXA1 protein expression was significantly increased in high-risk HPV squamous cell carcinoma of penis samples independently of the subtype of penile squamous cell carcinoma compared to the HPV negative squamous cell carcinoma of penis samples. So, probably ANXA1 might have an oncogenic role in penile cancer with high-risk HPVs.

HPV induces cervical cancer through uncontrolled G1-S transition. The E6 and E7 proteins of high-risk HPV inhibit p53 and pRb proteins, cell cycle regulatory proteins that control G1-S transition [46]. p16^INK4a^ (p16) is a protein belonging to the inhibitors of cyclin-dependent kinase (CDK) 4 family (INK4a family). The inactivation of pRb by E7 causes p16 overexpression as p16 is regulated by negative feedback of pRb [47]. Increased p16 expression has been observed in cancer samples of cervix [48], penis [49], head and neck [50], oral [51] and the anorectal region [52] when positive for high-risk HPVs and its overexpression was found to be a reliable marker for high-risk HPV in penile carcinoma [53]. p16 protein expression was significantly higher in penile carcinoma samples positive for high-risk HPVs independently of the subtype of penile squamous cell carcinoma compared to penile carcinoma HPV negative samples in our study. Some studies focused on p16 alterations in penile cancer, but with different emphases. One study found an overexpression of p16 in 29% of penile carcinomas, especially in connection with HPV infection [54]. Prowse et al. detected p16 overexpression in 46% of penile SCCs, which was significantly associated with HPV infection [49]. However, Senba et al. described p16 overexpression in an equal amount of HPV-positive and HPV-negative penile carcinomas from Kenya [55]. Based in our data, we suggested the p16 could be a marker for penile carcinoma, confirming the diagnosis of malignant penile lesions with high-risk HPVs corroborating with previous studies with the same type of cancer [49], [53], [56].

This study identified two overexpressed genes, *ANXA1* and *p16*, in penile squamous cell carcinoma positive for high-risk HPVs. To the best of our knowledge this report is the first to describe ANXA1 protein overexpression in penile carcinoma with high-risk HPV independently of the subtype. These genes are associated with various physiological processes including cellular differentiation, cell proliferation and signal transduction, suggesting that they have an important role in penile carcinogenesis. However, additional studies are required in order to elucidate their specific role in penile cancer with high-risk HPV.
